# Predictive Model for Occurrence of Febrile Neutropenia after Chemotherapy in Patients with Diffuse Large B-Cell Lymphoma: A Multicenter, Retrospective, Observational Study

**DOI:** 10.3390/hematolrep16010008

**Published:** 2024-02-07

**Authors:** Masaya Morimoto, Yuma Yokoya, Kikuaki Yoshida, Hideki Kosako, Yoshikazu Hori, Toshiki Mushino, Shinobu Tamura, Reiko Ito, Ryosuke Koyamada, Takuya Yamashita, Shinichiro Mori, Nobuyoshi Mori, Sachiko Ohde

**Affiliations:** 1 Department of Hematology/Oncology, Kinan Hospital, Wakayama 646-8588, Japan; 2 Department of Hematology/Oncology, Wakayama Medical University, Wakayama 641-8509, Japan; 3 Department of Hematology/Oncology, St. Luke’s International Hospital, Tokyo 104-0044, Japan; 4 Public Health, St. Luke’s International University, Tokyo 104-0044, Japan; 5 Department of Emergency and Intensive Care Medicine, Wakayama Medical University, Wakayama 641-8509, Japan; 6 Department of Infectious Diseases, St. Luke’s International Hospital, Tokyo 104-0044, Japan

**Keywords:** febrile neutropenia, chemotherapy, diffuse large B-cell lymphoma, outcomes, multivariate logistic regression model

## Abstract

Febrile neutropenia (FN) is a major concern in patients undergoing chemotherapy for diffuse large B-cell lymphoma (DLBCL); however, the overall risk of FN is difficult to assess. This study aimed to develop a model for predicting the occurrence of FN in patients with DLBCL. In this multicenter, retrospective, observational analysis, a multivariate logistic regression model was used to analyze the association between FN incidence and pretreatment clinical factors. We included adult inpatients and outpatients (aged ≥ 18 years) diagnosed with DLBCL who were treated with chemotherapy. The study examined 246 patients. Considering FN occurring during the first cycle of chemotherapy as the primary outcome, a predictive model with a total score of 5 points was constructed as follows: 1 point each for a positive hepatitis panel, extranodal involvement, and a high level of soluble interleukin-2 receptor and 2 points for lymphopenia. The area under the receiver operating characteristic curve of this model was 0.844 (95% confidence interval: 0.777–0.911). Our predictive model can assess the risk of FN before patients with DLBCL start chemotherapy, leading to better outcomes.

## 1. Introduction

Diffuse large B-cell lymphoma (DLBCL) is the most common subtype of lymphoma [1]. The cyclophosphamide, doxorubicin, vincristine, and prednisone (CHOP) regimen with added rituximab (R-CHOP) currently serves as a standard treatment for DLBCL. Chemotherapy-induced neutropenia and febrile neutropenia (FN) are common serious clinical conditions that occur during this chemotherapy regimen. FN is defined as an absolute neutrophil count of <0.5 × 10^9^/L, or <1.0 × 10^9^/L predicted to fall below 0.5 × 10^9^/L within 48 h, with fever or clinical signs of sepsis [2]. The European Society for Medical Oncology (ESMO) defines fever in this setting as a rise in axillary temperature to >38.5 °C sustained for at least one hour. Patients with FN require immediate antibiotic therapy because the infection can progress rapidly. Prolonged neutropenia and FN can delay the delivery of cancer treatment as well as reduce treatment effectiveness. A decreased dose intensity is associated with impaired outcomes in patients with DLBCL treated with chemotherapy [3]. Thus, prophylaxis and FN management are necessary to maintain planned doses of chemotherapy. Although the use of granulocyte colony-stimulating factor (G-CSF) can help reduce the risk of developing neutropenic events, some patients experience serious complications related to FN. In a study evaluating patterns of G-CSF usage and FN among patients with DLBCL treated with CHOP or R-CHOP regimens (CALGB 9793; ECOG-SWOG 4494), G-CSF use was significantly more common in the older population (age > 65 years) [4]. However, identification of patients who would benefit from its use in clinical settings remains challenging.

The American Society of Clinical Oncology and European Organization for Research and Treatment of Cancer recommend primary prophylaxis with G-CSF for patients undergoing chemotherapy regimens that pose a high risk of FN (≥20%) or an intermediate risk of FN (10–20%), depending on the patients’ individual risk factors [2,5]. According to recent studies, the risk of FN with CHOP-like regimens is 9.0–15.2% [6,7,8], which is classified as an intermediate risk. When using intermediate-risk chemotherapy regimens, patient-related risk factors must be evaluated to determine the appropriate FN treatment.

Several retrospective studies [9,10,11] and a systematic review [12] have identified the following possible patient-related risk factors for FN in patients with lymphoma: advanced age; poor performance status; advanced disease; comorbid renal, cardiovascular, or liver diseases; low baseline blood cell counts; and low serum albumin, abnormal bone marrow, and increased lactate dehydrogenase levels. However, little is known regarding the effects of these risk factors in patients with DLBCL. Assigning weights to these risk factors and determining their individual importance to guide the use of G-CSF in patients with DLBCL are difficult. If the causal association between baseline characteristics or disease status of a patient and FN incidence can be determined, appropriate interventions can be introduced in time to prevent FN onset.

More than half of the patients who develop FN experience an episode during their first cycle of chemotherapy [9,10]. FN development during the first cycle affects the timing and dosing of the subsequent cycles of chemotherapy and prophylactic management. Therefore, prediction of FN in the first cycle based on pretherapy risk factors should improve the overall prognosis of patients with DLBCL.

In this study, we aimed to develop a predictive model for FN development in the first cycle of the R-CHOP-like regimen treatment in treatment-naive patients with DLBCL.

## 2. Materials and Methods

### 2.1. Study Design and Patients

In this retrospective, multicenter cohort study, we reviewed the medical records of all adult inpatients and outpatients (aged ≥ 18 years) diagnosed with DLBCL who were treated with chemotherapy at Kinan Hospital (Wakayama, Japan) between 1 January 2010, and 31 December 2021, and at St. Luke’s International Hospital (Tokyo, Japan) between 1 January 2010, and 31 December 2018. Patients transferred to our hospital after starting treatment at other hospitals and those treated with rituximab only were excluded from this study.

As this study was conducted using existing data retrieved from medical records, the need for obtaining informed consent for participation was waived. This study was approved by the Research Ethics Committee of each hospital (Kinan Hospital reference number: 229, St. Luke’s International Hospital reference number: 19-R106). This study was conducted in accordance with the tenets of the Declaration of Helsinki.

### 2.2. Data Collection

All parameters evaluated were selected considering clinical insights and information from previous studies [9,10,11,12]. Data on patients’ demographics (age, sex, height, weight, and body mass index), disease status (pathological diagnosis, Ann Arbor stage, bone marrow infiltration, and extranodal involvement), complications (diabetes mellitus, viral hepatitis (without cirrhosis), chronic kidney disease, cardiac disease, chronic obstructive lung disease, and history of malignant diseases), and blood parameters (albumin level, total bilirubin level, creatinine level, estimated glomerular filtration rate, lactate dehydrogenase level, C-reactive protein level, soluble interleukin-2 receptor (sIL2R) level, white blood cell count, absolute neutrophil count (ANC), absolute lymphocyte count, hemoglobin level, and platelet count) were collected. Hepatitis C virus (HCV) infection was confirmed using a serological test for HCV antibody, and hepatitis B virus (HBV) infection was confirmed using a test for the surface antigen of hepatitis B (HBsAg), whereas previous infections were confirmed using tests for the surface antibody of hepatitis B (anti-HBs) or the core antibody (anti-HBc) without a history of immunization. If the result of any of these tests was positive, the patient was classified as having a “positive hepatitis panel”. Although active hepatitis B (HepB), chronic HepB, inactive HepB, and hepatitis C (HepC) are distinct disease entities, we grouped these under the term “positive hepatitis panel”, aiming to assess the collective risk and impact. The following treatment data were also collected: type of chemotherapy regimen, relative dose intensity, and use of prophylactic antibiotics and G-CSF. Patients treated with only the initial treatment (R-CHOP-like regimens) throughout the course were included in the R-CHOP-like therapy group, whereas those who underwent the salvage treatment, which caused severe myelosuppression, for lymphoma relapse were included in the salvage therapy group. The “RCHOP-like” group consists of patients who received R-CHOP as their initial treatment and did not experience a relapse, thus undergoing only the initial therapy. The “Salvage therapy” group includes patients who had experienced a relapse at least once and required salvage chemotherapy after their initial R-CHOP treatment. By categorizing patients in this manner, we aimed to distinguish between those who remained relapse-free after the initial therapy and those who required additional interventions due to relapse. As the intensity of chemotherapy is clearly related to the risk of developing FN, we evaluated the data of patients in the R-CHOP-like and salvage therapy groups separately.

### 2.3. Study Endpoints

The primary endpoint was the FN incidence during the first cycle of chemotherapy for DLBCL. The secondary endpoint was the FN incidence during any cycle of chemotherapy for DLBCL. FN was defined as the presence of an axillary body temperature ≥ 37.5 °C in addition to neutropenia (ANC < 500 cells/mm^3^ or ANC expected to decrease to <500 cells/mm^3^ during the next 48 h).

### 2.4. Statistical Analyses

Descriptive statistics were used to summarize the baseline measurements. Continuous and categorical variables are presented as medians (interquartile ranges) and numbers (%), respectively. To construct the predictive model, the hematological parameters and other continuous variables were converted into binary variables using clinically important values as cutoff points. We used the chi-squared (χ^2^) test to compare parameters between patients with and without FN. Univariate and multivariate logistic regression models were used to analyze the risk factors associated with FN onset. Considering the rule of 10 events per variable [13,14,15] and our sample size (37 + 157), we limited the number of predictors to four to prevent model overfitting. All candidate predictors with a *p*-value < 0.2 in the univariate analysis were included in a backward stepwise logistic regression model, and a *p*-value of 0.05 was used as the cutoff point for final entry or removal. The scores for each predictor were obtained using the beta coefficients of the final model. A receiver operating characteristic curve was drawn, and the area under the curve (AUC) was obtained. For internal validation, bootstrapping was performed with 1000 iterations to simulate unbiased outcomes. All statistical analyses were performed using Stata version 17.0 (StataCorp., College Station, TX, USA).

## 3. Results

In total, 134 patients from Kinan Hospital and 112 patients from St. Luke’s International Hospital were included. Most patients were Japanese (Japanese, 240; Caucasian, 5; Hispanic, 1). The baseline demographic and disease characteristics are summarized according to the intensity of the chemotherapy regimen in Table 1. Of the 246 patients with DLBCL, 194 received only R-CHOP-like therapy and 52 received at least one salvage therapy. The intensity of chemotherapy was significantly associated with FN incidence during any cycle of chemotherapy (R-CHOP-like therapy: 33.0% [64/194], salvage therapy: 70.6% [36/52], *p* < 0.001). Thus, the results were analyzed according to the intensity of the chemotherapy regimen. In this study, our analysis focused specifically on 194 patients who received R-CHOP-like therapy. The ages of the patients ranged from 25 to 100 years, and the median age was 73.0 (63.0–80.0) years. More women (56%) than men (44%) were enrolled. Diabetes (16%) and cardiac disease (17%) were the most common comorbidities. None of the patients tested positive for HIV antibodies. More than half of the patients had advanced disease (Ann Arbor stages I–II, 45%; III–IV, 55%). Moreover, 15% of those patients had bone marrow infiltration, and extranodal involvement was present in half of the patients. Furthermore, 17% of the patients tested positive for HBV, but none required antiviral prophylaxis because they were HBV-DNA negative, and all had either previously been infected with HBV or were post-vaccinated.

Univariate analysis results for factors associated with FN development in the first cycle of R-CHOP-like therapy are shown in Table 2. Of the 194 patients in the R-CHOP-like therapy group, 69 received G-CSF and 125 did not. Administration of G-CSF to patients was at the physician’s discretion. Additionally, 15 of the 125 (11.2%) patients who did not receive G-CSF and 23 of the 69 (33.3%) patients who did receive G-CSF developed FN (Figure S1). Patients who were treated with R-CHOP-like regimens did not receive antimicrobial prophylaxis. The associations between the variables and FN incidence during any cycle of chemotherapy (secondary endpoint) are shown in Table S1. Factors associated with FN development in the salvage regimen group are shown in Tables S2–S4. According to the univariate analysis, a history of pulmonary disease, six laboratory parameters (platelet count, lymphocyte count, albumin level, lactate dehydrogenase level, C-reactive protein level, and sIL2R level), three factors related to lymphoma (Ann Arbor stage, extranodal involvement, and bone marrow infiltration), and a positive hepatitis panel were selected as candidate predictors of the occurrence of FN during the first cycle of R-CHOP-like therapy. Backward stepwise logistic regression analysis of all candidate predictors showed that a positive hepatitis panel, extranodal involvement, low lymphocyte count (lymphopenia), and elevated sIL2R levels (all *p* < 0.05) were significant prognostic predictors. The results of this analysis for FN incidence in the first cycle of R-CHOP-like chemotherapy are shown in Table 3.

**Figure 1:**
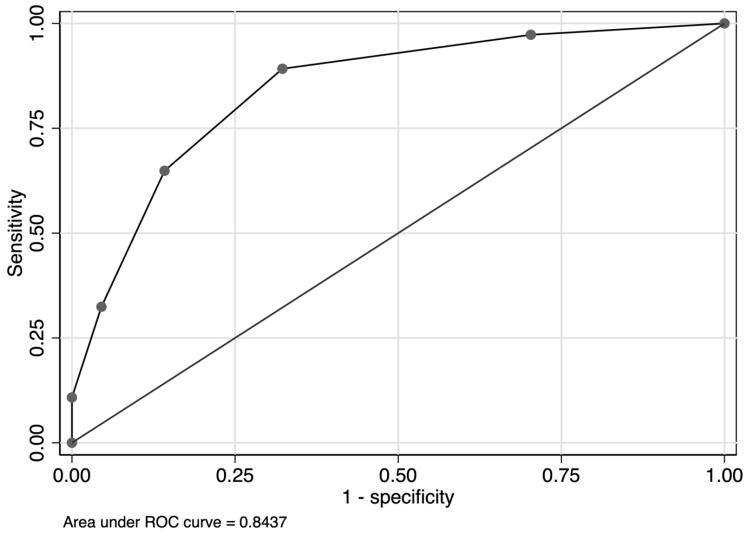
ROC curve of the prediction score for developing FN during the first cycle of chemotherapy for patients treated with the R-CHOP-like regimen. Area under the curve (95% confidence interval) = 0.844 (0.777–0.911). FN, febrile neutropenia; R-CHOP, cyclophosphamide, doxorubicin, vincristine, and prednisone with added rituximab; ROC, receiver operating characteristic.

**Figure 2:**
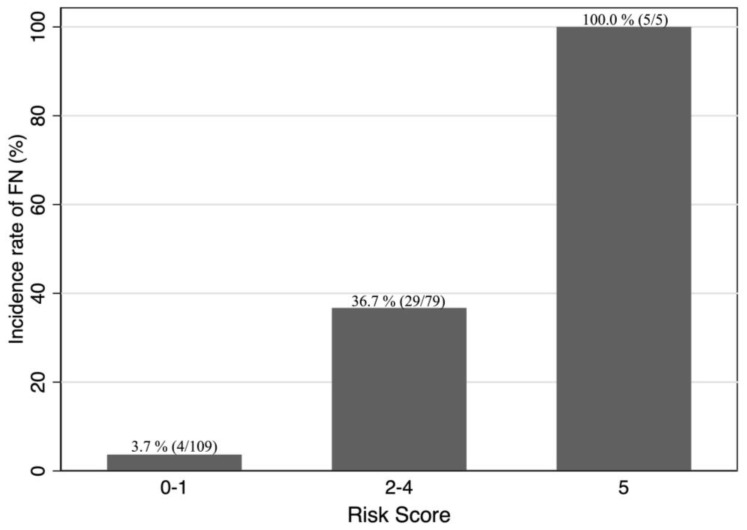
Incidence rate of FN during the first cycle of chemotherapy for patients treated with the R-CHOP-like regimen based on prediction scores. For risk scores 0–1, out of 109 patients, 4 (3.7%) had FN. In the 2–4 score group, 29 out of 79 patients (36.7%) experienced FN. All 4 patients with a score of 5 (100%) developed FN. FN, febrile neutropenia; R-CHOP, cyclophosphamide, doxorubicin, vincristine, and prednisone with added rituximab.

For factors associated with FN development during the first cycle of chemotherapy, the scores for each predictor were obtained according to the beta coefficients of the factors as follows: lymphopenia, 2 points; high sIL2R level, 1 point; extranodal involvement, 1 point; and a positive hepatitis panel, 1 point. We calculated the sum of the scores for each patient and created a receiver operating characteristic curve (Figure 1). The AUC (95% confidence interval (CI)) of this model was 0.844 (0.777–0.911). The distribution of the prognostic scores and FN incidence for each score group are shown in Figure 2 and Figure S2. When the cutoff value for this score was 2 points, the sensitivity and specificity of the model were 89.2% and 67.7%, respectively. Moreover, the observed beta coefficient and bootstrapped validation results were identical (Table 4 and Table S5). All simulation data indicated that the model had high internal validity.

## 4. Discussion

This retrospective, observational study identified potential risk factors for FN during chemotherapy in patients with DLBCL. Among the 246 patients who received chemotherapy, 100 developed FN at least once during the treatment cycles, including 46 (46.0%) who developed FN during the first treatment cycle. These data were consistent with those of a previous report [16] and indicate the importance of evaluating the possibility of FN during the first chemotherapy session. The four predictors identified in the multivariate logistic regression analysis were lymphopenia, elevated sIL2R level, extranodal involvement, and a positive hepatitis panel. These were included in our predictive model, which can be used to calculate a score indicating the risk of developing FN in patients with DLBCL undergoing chemotherapy. A total score of 0–1 points indicates a low probability of developing FN. Having this information before starting chemotherapy is beneficial for determining the length of hospitalization and planning outpatient treatment.

Although liver cirrhosis is associated with severe infection, owing to the impairment of innate immune function caused by the disease [17], no previous study has shown an association between hepatitis viral status and FN occurrence. This study indicated that a positive hepatitis panel is a significant risk factor for FN occurrence in the first cycle of chemotherapy in patients with DLBCL (odds ratio (OR) [95% CI]: 4.85 [1.85–12.74]) (Table 3). This finding has important clinical implications.

Positivity for HCV and anti-HBc was also significantly associated with FN development in the present study. Multivariate analysis using HCV and anti-HBc factors showed that HCV-positivity was significantly associated with FN development (Table S6). Anti-HBc-positivity in patients who are HBsAg(–) and lack anti-HBs is referred to as “isolated anti-HBc” [18] and is considered to be indicative of a functional cure of HBV infection. Patients with HBV-DNA in the blood, or replication-competent HBV-DNA in the liver, who are HBsAg(–)/anti-HBc(+) remain infectious and harbor an occult, seropositive HBV infection [19]. Of the 194 patients in the R-CHOP-like therapy group in this study, 10 (5.2%) had isolated anti-HBc. However, no significant association was observed between isolated anti-HBc and FN development in the multivariate analysis (OR [95% CI]: 2.02 [0.36–11.32]). The prevalence of HCV infection in patients with non-Hodgkin’s lymphoma (NHL) is higher than that in the general population [20]. Furthermore, HCV infection can increase the risk of developing NHL by 2.5-fold [21]. However, the mechanisms underlying the development of HCV-associated lymphoma remain controversial. A recent meta-analysis indicated the poor prognosis and distinct clinical characteristics of HCV-associated NHL, particularly in patients with DLBCL [22]. However, no association with the onset of FN was mentioned. To the best of our knowledge, no previous study has shown a correlation between hepatitis viral status and FN development. Regarding the association between HCV and lymphoid neoplasms, experimental data suggest multistep processes [23,24,25]. However, there are no data on the association between HCV and FN development. Therefore, future studies are required to clarify the correlation between HCV development and FN.

Extranodal involvement affects the prognosis of patients with DLBCL and is listed as a risk factor on the International Prognostic Index [26]. Elevated sIL2R levels before treatment have also been associated with a poor prognosis in patients with NHL, including DLBCL [27,28]. Extranodal involvement and elevated sIL2R levels are associated with the DLBCL burden. A recent study investigated the molecular and cellular roles of G-CSF receptor signaling in chemotherapy-induced neutropenia during chemotherapy in patients with DLBCL and suggested that a high burden of DLBCL changes the bone marrow environment and the G-CSF receptor signaling pathway [29]. Some cytokines secreted by lymphoma cells alter the bone marrow environment, causing chemotherapy-induced neutropenia and FN during chemotherapy. This may partially underlie the occurrence of FN events during the first cycle of chemotherapy, when the burden of DLBCL is significant.

Lymphopenia was an important prognostic factor for the entire population of this study (Table 3 and Table S4). Lymphopenia can be caused by several conditions, such as congenital immunodeficiency diseases [30], malnutrition [31], malignancies [32], systemic autoimmune diseases [33], and infections [34]. A prospective study showed that lymphopenia is associated with a high risk of hospitalization and infection-related mortality among patients with cancer [35]. Although the causality of this correlation is unknown, lymphopenia is clinically a crucial factor in the development of infectious diseases and FN in patients receiving chemotherapy. A previous study showed that early development of lymphopenia, on day 5 after chemotherapy administration, is an independent risk factor for FN development [36]. Another study indicated that a low CD4 count is an independent risk factor for FN and early mortality in patients receiving cytotoxic chemotherapy [37]. In the present study, low white blood cell count, low ANC, anemia, and thrombocytopenia were not significantly associated with FN development. Only the association between lymphopenia and FN development was statistically significant. Therefore, further studies on the correlation of the neutrophil/lymphocyte ratio, CD4/CD8 ratio, and results of lymphocyte flow cytometry with FN development are warranted.

Advanced age (≥65 years) has been identified as a risk factor for FN development [38,39]. However, we did not find any evidence supporting this finding in this study. This may be attributed to the fact that the reduced chemotherapy dose administered to geriatric patients causes milder myelosuppression than that caused by the usual regimen. As Japan is a super-aged society, the median age of the patients in this study was 70 years. Thus, this study focused on FN development in older individuals.

This study has several limitations. First, the patients in the study population were selected from only two facilities: St. Luke’s International Hospital in the center of the capital city of Japan and Kinan Hospital in the countryside of Japan. However, the mean ages of the patients selected from each hospital were significantly different (*p* = 0.001), indicating that the study population was diverse. We performed bootstrapping for internal validation of the predictive model; however, this method is not sufficient, and external validation of the model in future studies is necessary. Additionally, some limitations regarding G-CSF prophylaxis were present. We analyzed the data of patients who received G-CSF prophylaxis for initial chemotherapy and those of patients who did not. However, prophylactic care was often performed at the discretion of the primary physician. In addition, some chemotherapy sessions included prophylaxis, while others for the same patients did not. Regarding the secondary outcome of this study, assessing prophylaxis as a potential risk factor for FN was challenging because the outcome was evaluated for each patient rather than for each treatment episode. Finally, we focused on FN onset and development, rather than on the frequency of infection-related admission or death. Although FN is associated with a longer hospitalization duration, higher costs, and mortality [40], we were unable to analyze the association between these factors in this study.

According to the guidelines on the appropriate use of G-CSF [2,5], the incidence rate of FN in patients undergoing myelosuppressive chemotherapy is 13–21%. In this study, the incidence rate of FN during the first cycle of chemotherapy was 18.7% (46/246), which is consistent with the results of previous studies. FN development during chemotherapy is significantly associated with a long-term increase in the risk of infections [41]. Furthermore, FN can delay the treatment schedule and lead to the early termination and reduction of chemotherapy doses, which are associated with high mortality rates. Therefore, preventing FN can improve the overall treatment outcomes of patients. Several studies have indicated that prophylaxis with G-CSF can reduce FN development [2,42]. However, routine administration of primary G-CSF prophylaxis to all patients undergoing chemotherapy is neither practical nor clinically appropriate, given the significant costs associated with this agent [43]. If our model can be used to differentiate patients who need G-CSF from those who do not, it may help optimize the treatment of lymphoma.

The predictive model for FN development during the first cycle of chemotherapy for DLBCL yielded an AUC indicating a relatively high level of accuracy (AUC = 0.844; 95% CI: 0.777–0.911). The incidence rate of FN was significantly low (3.7%) among patients whose total score calculated using this model was 0–1 points (Figure 2). The sensitivity of the model was 89.2% when the cutoff value of the score was 2 of 5 points. We believe that this model can be used as a tool to identify patients with a low probability of developing FN in the future.

## 5. Conclusions

In this study, we established a predictive model for the development of FN in patients with DLBCL based on four variables: a positive hepatitis panel, extranodal involvement, lymphopenia, and high sIL2R level. This model had a relatively high AUC, demonstrating its value in clinical settings. Our findings suggest that patients with a low score (0–1 points) in this model can be transferred from inpatient to outpatient care relatively earlier, without the need for G-CSF administration.

## Figures and Tables

**Table 1:** Demographic and clinical characteristics of patients according to the intensity of chemotherapy regimens.

	Total DLBCL	R-CHOP-like	Salvage Therapy
	*n* = 246	*n* = 194	*n* = 52
Age, years	72.0 (63.0–79.0)	73.0 (63.0–80.0)	69.5 (61.5–75.5)
Sex			
Female	146 (59%)	109 (56%)	37 (71%)
Male	100 (41%)	85 (44%)	15 (29%)
Comorbidity			
Diabetes	36 (15%)	32 (16%)	4 (8%)
Chronic kidney disease	14 (6%)	10 (5%)	4 (8%)
Cardiac disease	38 (15%)	33 (17%)	5 (10%)
Pulmonary disease	5 (2%)	4 (2%)	1 (2%)
Malignancy	31 (13%)	24 (12%)	7 (13%)
Ann Arbor stage			
I	39 (16%)	38 (20%)	1 (2%)
II	55 (22%)	48 (25%)	7 (13%)
III	44 (18%)	34 (18%)	10 (19%)
IV	108 (44%)	74 (38%)	34 (65%)
Bone marrow infiltration			
Yes	44 (18%)	30 (15%)	14 (27%)
No	202 (82%)	164 (85%)	38 (73%)
Extranodal involvement			
Yes	140 (57%)	102 (53%)	38 (73%)
No	106 (43%)	92 (47%)	14 (27%)
Baseline laboratory data			
eGFR < 60 mL/min/1.73 m^2^	61 (25%)	52 (27%)	9 (17%)
T-Bil > 1.0 g/dL	35 (15%)	27 (14%)	8 (16%)
Albumin < 3.5 g/dL	87 (36%)	71 (37%)	16 (31%)
LD > 222 IU/L	147 (60%)	109 (57%)	38 (73%)
CRP > 10 mg/dL	13 (6%)	10 (6%)	3 (6%)
sIL2R > 2000 U/mL	97 (40%)	72 (37%)	25 (48%)
WBC < 3.5 × 10^9^/L	29 (12%)	22 (11%)	7 (13%)
ANC < 1.5 × 10^9^/L	9 (4%)	8 (4%)	1 (2%)
ALC < 0.7 × 10^9^/L	54 (22%)	42 (22%)	12 (23%)
Hemoglobin < 12.0 g/dL	114 (46%)	92 (47%)	22 (42%)
Platelet count < 100 × 10^9^/L	32 (13%)	24 (12%)	8 (15%)
Hepatitis viral status			
Positive hepatitis panel	50 (20%)	40 (21%)	10 (19%)
HCV	15 (6%)	13 (7%)	2 (4%)
HBV	43 (17%)	33 (17%)	10 (19%)
HBsAg+	5 (2%)	3 (2%)	2 (4%)
anti-HBs+	27 (11%)	21 (11%)	6 (12%)
anti-HBc+	34 (14%)	28 (14%)	6 (12%)

Abbreviations: DLBCL, diffuse large B-cell lymphoma; R-CHOP, cyclophosphamide, doxorubicin, vincristine, and prednisone with rituximab; eGFR, estimated glomerular filtration rate; T-Bil, total bilirubin; LD, lactate dehydrogenase; CRP, C-reactive protein; sIL2R, soluble interleukin-2 receptor; WBC, white blood cell; ANC, absolute neutrophil count; ALC, absolute lymphocyte count; HCV, hepatitis C virus; HBV, hepatitis B virus; anti-HBsAg, hepatitis B surface antigen; HBs, hepatitis B surface antibody; anti-HBc, hepatitis B core antibody.

**Table 2:** Univariate analysis of factors associated with FN incidence during the first cycle of chemotherapy for patients treated with the R-CHOP-like regimen.

	Developed FN during the First Therapy Cycle	Did Not Develop FN during the First Therapy Cycle	*p*-Value
	*n* = 37	*n* = 157	
Age ≥ 65 years	26 (70%)	112 (71%)	0.90
Sex: male	21 (57%)	88 (56%)	0.94
Comorbidity			
Diabetes	4 (11%)	28 (18%)	0.30
Chronic kidney disease	2 (5%)	8 (5%)	0.94
Cardiac disease	7 (19%)	26 (17%)	0.73
Pulmonary disease	2 (5%)	2 (1%)	0.11
Malignancy	6 (16%)	18 (11%)	0.43
Baseline laboratory data			
WBC < 3.5 × 10^9^/L	5 (14%)	17 (11%)	0.64
Hemoglobin < 12.0 g/dL	20 (54%)	72 (46%)	0.37
Platelet < 100 × 10^9^/L	11 (30%)	13 (8%)	<0.001
ANC < 1.5 × 10^9^/L	2 (5%)	6 (4%)	0.67
ALC < 0.7 × 10^9^/L	20 (54%)	22 (14%)	<0.001
T-Bil > 1.0 g/dL	6 (16%)	21 (14%)	0.73
Albumin < 3.5 g/dL	22 (63%)	49 (31%)	<0.001
LD > 222 IU/L	27 (75%)	82 (53%)	0.014
CRP > 10 mg/dL	5 (14%)	5 (4%)	0.02
eGFR < 60 mL/min/1.73 m^2^	13 (35%)	39 (25%)	0.2
sIL2R > 2000 U/mL	26 (70%)	46 (29%)	<0.001
Ann Arbor stage: Advanced (III–IV)	30 (83%)	78 (50%)	<0.001
Extranodal involvement	27 (73%)	75 (48%)	0.006
Bone marrow infiltration	12 (32%)	18 (11%)	0.002
Hepatitis viral status			
Positive hepatitis panel	16 (43%)	24 (15%)	<0.001
HCV	8 (22%)	5 (3%)	<0.001
HBV	11 (30%)	22 (14%)	0.022
HBsAg+	1 (3%)	2 (1%)	0.53
anti-HBs+	6 (16%)	15 (10%)	0.24
anti-HBc+	11 (30%)	17 (11%)	0.003
G-CSF administration	23 (62%)	46 (29%)	<0.001

Abbreviations: FN, febrile neutropenia; R-CHOP, cyclophosphamide, doxorubicin, vincristine, and prednisone with rituximab; WBC, white blood cell; ANC, absolute neutrophil count; ALC, absolute lymphocyte count; T-Bil, total bilirubin; LD, lactate dehydrogenase; CRP, C-reactive protein; eGFR, estimated glomerular filtration rate; sIL2R, soluble interleukin-2 receptor; HCV, hepatitis C virus; HBV, hepatitis B virus; HBsAg, hepatitis B surface antigen; anti-HBs, hepatitis B surface antibody; anti-HBc, hepatitis B core antibody.

**Table 3:** Multivariate logistic regression of risk factors associated with FN incidence during the first cycle of chemotherapy for patients treated with the R-CHOP-like regimen.

				*n* = 191
	Odds Ratio [95% CI]	β Coefficient [95% CI]	Score	*p*-Value
ALC < 0.7 × 10^9^/L	6.32 [2.51–15.92]	1.84 [0.92–2.77]	2	<0.001
sIL2R > 2000 U/mL	3.05 [1.21–7.67]	1.12 [0.19–2.04]	1	0.018
Extranodal involvement	2.94 [1.08–7.99]	1.08 [0.08–2.08]	1	0.034
Positive hepatitis panel	4.85 [1.85–12.74]	1.58 [0.61–2.54]	1	0.001

Abbreviations: FN, febrile neutropenia; R-CHOP, cyclophosphamide, doxorubicin, vincristine, and prednisone with rituximab; CI, confidence interval; ALC, absolute lymphocyte count; sIL2R, soluble interleukin-2 receptor.

**Table 4:** Bootstrap validation of the prediction model for FN occurrence during the first cycle of chemotherapy for patients treated with the R-CHOP-like regimen and comparison with the original model.

			*n* = 191
	Logistic Regression β Coefficient [95% CI]	Bootstrapped β Coefficient [95% CI]	Score
ALC < 0.7 × 10^9^/L	1.84 [0.92–2.77]	1.97 [0.81–2.88]	2
sIL2R > 2000 U/mL	1.12 [0.19–2.04]	1.17 [0.09–2.14]	1
Extranodal involvement	1.08 [0.08–2.08]	1.18 [0.02–2.14]	1
Positive hepatitis panel	1.58 [0.61–2.54]	1.68 [0.55–2.61]	1

Abbreviations: FN, febrile neutropenia; R-CHOP, cyclophosphamide, doxorubicin, vincristine, and prednisone with rituximab; CI, confidence interval; ALC, absolute lymphocyte count; sIL2R, soluble interleukin-2 receptor.

## Data Availability

The datasets analyzed during the current study are available from the corresponding author on reasonable request.

## Supplementary Materials

The following supporting information can be downloaded at: https://www.mdpi.com/article/10.3390/hematolrep16010008/s1, Figure S1. Differences in FN incidence between patients who received G-CSF during the first cycle of R-CHOP-like therapy and those who did not; Figure S2. Incidence rate of FN during the first cycle of chemotherapy for patients treated with the R-CHOP-like regimen based on each prediction score; Table S1. Univariate analysis of factors associated with febrile neutropenia incidence during any cycle for patients treated with the R-CHOP-like regimen; Table S2. Univariate analysis of factors associated with febrile neutropenia incidence during the first cycle among patients in the salvage therapy group; Table S3. Univariate analysis of factors associated with febrile neutropenia incidence during any cycle among patients in the salvage therapy group; Table S4. Multivariate logistic regression analysis of risk factors associated with febrile neutropenia incidence during any cycle among patients treated with the R-CHOP-like regimen; Table S5. Bootstrap validation of the predictive model for febrile neutropenia occurrence during any cycle among patients treated with the R-CHOP-like regimen and comparison with the original model; Table S6. Multivariate logistic regression of risk factors associated with febrile neutropenia incidence during the first cycle of chemotherapy among patients treated with the R-CHOP-like regimen, with a more detailed evaluation of the viral hepatitis status.

## References

[1] Teras L.R., DeSantis C.E., Cerhan J.R., Morton L.M., Jemal A., Flowers C.R. (2016). 2016 US lymphoid malignancy statistics by World Health Organization subtypes. CA Cancer J. Clin..

[2] Aapro M.S., Bohlius J., Cameron D.A., Dal Lago L., Donnelly J.P., Kearney N., Lyman G.H., Pettengell R., Tjan-Heijnen V.C., Walewski J. (2011). 2010 update of EORTC guidelines for the use of granulocyte-colony stimulating factor to reduce the incidence of chemotherapy-induced febrile neutropenia in adult patients with lymphoproliferative disorders and solid tumours. Eur. J. Cancer.

[3] Pettengell R., Schwenkglenks M., Leonard R., Bosly A., Paridaens R., Constenla M., Szucs T.D., Jackisch C., Impact of Neutropenia in Chemotherapy-European Study Group (INC-EU) (2008). Neutropenia occurrence and predictors of reduced chemotherapy delivery: Results from the INC-EU prospective observational European neutropenia study. Support. Care Cancer.

[4] Morrison V.A., Weller E.A., Habermann T.M., Li S., Fisher R.I., Cheson B.D., Peterson B.A. (2017). Patterns of growth factor usage and febrile neutropenia among older patients with diffuse large B-cell non-Hodgkin lymphoma treated with CHOP or R-CHOP: The Intergroup experience (CALGB 9793; ECOG-SWOG 4494). Leuk. Lymphoma.

[5] Smith T.J., Bohlke K., Lyman G.H., Carson K.R., Crawford J., Cross S.J., Goldberg J.M., Khatcheressian J.L., Leighl N.B., Perkins C.L. (2015). Recommendations for the use of WBC growth factors: American Society of Clinical Oncology clinical practice guideline update. J. Clin. Oncol..

[6] Vitolo U., Trněný M., Belada D., Burke J.M., Carella A.M., Chua N., Abrisqueta P., Demeter J., Flinn I., Hong X. (2017). Obinutuzumab or rituximab plus cyclophosphamide, doxorubicin, vincristine, and prednisone in previously untreated diffuse large B-cell lymphoma. J. Clin. Oncol..

[7] Nowakowski G.S., Chiappella A., Gascoyne R.D., Scott D.W., Zhang Q., Jurczak W., Özcan M., Hong X., Zhu J., Jin J. (2021). ROBUST: A phase III study of lenalidomide plus R-CHOP versus placebo plus R-CHOP in previously untreated patients with abc-type diffuse large B-cell lymphoma. J. Clin. Oncol..

[8] Younes A., Sehn L.H., Johnson P., Zinzani P.L., Hong X., Zhu J., Patti C., Belada D., Samoilova O., Suh C. (2019). Randomized phase III trial of ibrutinib and rituximab plus cyclophosphamide, doxorubicin, vincristine, and prednisone in non-germinal center B-cell diffuse large B-cell lymphoma. J. Clin. Oncol..

[9] Lyman G.H., Delgado D.J. (2003). Risk and timing of hospitalization for febrile neutropenia in patients receiving CHOP, CHOP-R, or CNOP chemotherapy for intermediate-grade non-Hodgkin lymphoma. Cancer.

[10] Lyman G.H., Morrison V.A., Dale D.C., Crawford J., Delgado D.J., Fridman M., OPPS Working Group, ANC Study Group (2003). Risk of febrile neutropenia among patients with intermediate-grade non-Hodgkin’s lymphoma receiving CHOP chemotherapy. Leuk. Lymphoma.

[11] Rabinowitz A.P., Weiner N.J., Tronic B.S., Fridman M., Liberman R.F., Delgado D.J. (2006). Severe neutropenia in CHOP occurs most frequently in cycle 1: A predictive model. Leuk. Lymphoma.

[12] Lyman G.H., Abella E., Pettengell R. (2014). Risk factors for febrile neutropenia among patients with cancer receiving chemotherapy: A systematic review. Crit. Rev. Oncol. Hematol..

[13] Concato J., Peduzzi P., Holford T.R., Feinstein A.R. (1995). Importance of events per independent variable in proportional hazards analysis I. Background, goals, and general strategy. J. Clin. Epidemiol..

[14] Peduzzi P., Concato J., Feinstein A.R., Holford T.R. (1995). Importance of events per independent variable in proportional hazards regression analysis II. Accuracy and precision of regression estimates. J. Clin. Epidemiol..

[15] Peduzzi P., Concato J., Kemper E., Holford T.R., Feinstein A.R. (1996). A simulation study of the number of events per variable in logistic regression analysis. J. Clin. Epidemiol..

[16] Choi Y.W., Jeong S.H., Ahn M.S., Lee H.W., Kang S.Y., Choi J.H., Jin U.R., Park J.S. (2014). Patterns of neutropenia and risk factors for febrile neutropenia of diffuse large B-cell lymphoma patients treated with rituximab-CHOP. J. Korean Med. Sci..

[17] Irvine K.M., Ratnasekera I., Powell E.E., Hume D.A. (2019). Causes and consequences of innate immune dysfunction in cirrhosis. Front. Immunol..

[18] Moretto F., Catherine F.X., Esteve C., Blot M., Piroth L. (2020). Isolated anti-HBc: Significance and management. J. Clin. Med..

[19] Raimondo G., Locarnini S., Pollicino T., Levrero M., Zoulim F., Lok A.S., Taormina Workshop on Occult HBV Infection Faculty Members (2019). Update of the statements on biology and clinical impact of occult hepatitis B virus infection. J. Hepatol..

[20] Gisbert J.P., García-Buey L., Arranz R., Blas C., Pinilla I., Khorrami S., Acevedo A., Borque M.J., Pajares J.M., Fernández-Rañada J.M. (2004). The prevalence of hepatitis C virus infection in patients with non-Hodgkin’s lymphoma. Eur. J. Gastroenterol. Hepatol..

[21] Dal Maso L., Franceschi S. (2006). Hepatitis C virus and risk of lymphoma and other lymphoid neoplasms: A meta-analysis of epidemiologic studies. Cancer Epidemiol. Biomarkers Prev..

[22] Zhang M., Gao F., Peng L., Shen L., Zhao P., Ni B., Hou J., Huang H. (2021). Distinct clinical features and prognostic factors of hepatitis C virus-associated non-Hodgkin’s lymphoma: A systematic review and meta-analysis. Cancer Cell Int..

[23] Marcucci F., Mele A. (2011). Hepatitis viruses and non-Hodgkin lymphoma: Epidemiology, mechanisms of tumorigenesis, and therapeutic opportunities. Blood.

[24] Peveling-Oberhag J., Arcaini L., Hansmann M.L., Zeuzem S. (2013). Hepatitis C-associated B-cell non-Hodgkin lymphomas. Epidemiology, molecular signature and clinical management. J. Hepatol..

[25] Ferri C., Sebastiani M., Giuggioli D., Colaci M., Fallahi P., Piluso A., Antonelli A., Zignego A.L. (2015). Hepatitis C virus syndrome: A constellation of organ- and non-organ specific autoimmune disorders, B-cell non-Hodgkin’s lymphoma, and cancer. World J. Hepatol..

[26] Vokes E.E., Weichselbaum R.R., Lippman S.M., Hong W.K. (1993). Head and neck cancer. N. Engl. J. Med..

[27] Goto N., Tsurumi H., Goto H., Shimomura Y.I., Kasahara S., Hara T., Yasuda I., Shimizu M., Murakami N., Yoshikawa T. (2012). Serum soluble interleukin-2 receptor (sIL-2R) level is associated with the outcome of patients with diffuse large B cell lymphoma treated with R-CHOP regimens. Ann. Hematol..

[28] Umino K., Fujiwara S.I., Minakata D., Yamamoto C., Meguro A., Matsuyama T., Sato K., Ohmine K., Izumi T., Muroi K. (2019). Prognostic impact of serum soluble interleukin-2 receptor level at diagnosis in elderly patients with diffuse large B-cell lymphoma treated with R-CHOP. Leuk. Lymphoma.

[29] Kim D.Y., Nam J., Chung J.S., Jeon B.E., Lee J.H., Jo J.C., Kim S.W., Shin H.J. (2022). Predictive parameters of febrile neutropenia and clinical significance of G-CSF receptor signaling pathway in the development of neutropenia during R-CHOP chemotherapy with prophylactic pegfilgrastim in patients with diffuse large B-cell lymphoma. Cancer Res. Treat..

[30] Stephan J.L., Vlekova V., Le Deist F., Blanche S., Donadieu J., De Saint-Basile G., Durandy A., Griscelli C., Fischer A. (1993). Severe combined immunodeficiency: A retrospective single-center study of clinical presentation and outcome in 117 patients. J. Pediatr..

[31] Savino W. (2002). The thymus gland is a target in malnutrition. Eur. J. Clin. Nutr..

[32] Ray-Coquard I., Cropet C., Van Glabbeke M., Sebban C., Le Cesne A., Judson I., Tredan O., Verweij J., Biron P., Labidi I. (2009). Lymphopenia as a prognostic factor for overall survival in advanced carcinomas, sarcomas, and lymphomas. Cancer Res..

[33] Merayo-Chalico J., Gómez-Martín D., Piñeirúa-Menéndez A., Santana-de Anda K., Alcocer-Varela J. (2013). Lymphopenia as risk factor for development of severe infections in patients with systemic lupus erythematosus: A case-control study. QJM.

[34] Ahmad D.S., Esmadi M., Steinmann W.C. (2013). Idiopathic CD4 Lymphocytopenia: Spectrum of opportunistic infections, malignancies, and autoimmune diseases. Avicenna J. Med..

[35] Warny M., Helby J., Nordestgaard B.G., Birgens H., Bojesen S.E. (2018). Lymphopenia and risk of infection and infection-related death in 98,344 individuals from a prospective Danish population-based study. PLoS Med..

[36] Ray-Coquard I., Borg C., Bachelot T., Sebban C., Philip I., Clapisson G., Le Cesne A., Biron P., Chauvin F., Blay J.Y. (2003). Baseline and early lymphopenia predict for the risk of febrile neutropenia after chemotherapy. Br. J. Cancer.

[37] Borg C., Ray-Coquard I., Philip I., Clapisson G., Bendriss-Vermare N., Menetrier-Caux C., Sebban C., Biron P., Blay J.Y. (2004). CD4 Lymphopenia as a risk factor for febrile neutropenia and early death after cytotoxic chemotherapy in adult patients with cancer. Cancer.

[38] Nordvig J., Aagaard T., Daugaard G., Brown P., Sengeløv H., Lundgren J., Helleberg M. (2018). Febrile neutropenia and long-term risk of infection among patients treated with chemotherapy for malignant diseases. Open Forum Infect. Dis..

[39] Case D.C., Desch C.E., Kalman L.A., Vongkovit P., Mena R.R., Fridman M., Allen B. (2007). Community-based trial of R-CHOP and maintenance rituximab for intermediate- or high-grade non-Hodgkin lymphoma with first-cycle filgrastim for older patients. Clin. Lymphoma Myeloma.

[40] Lathia N., Isogai P.K., De Angelis C., Smith T.J., Cheung M., Mittmann N., Hoch J.S., Walker S. (2013). Cost-effectiveness of filgrastim and pegfilgrastim as primary prophylaxis against febrile neutropenia in lymphoma patients. J. Natl. Cancer Inst..

[41] Salar A., Haioun C., Rossi F.G., Duehrsen U., Pettengell R., Johnsen H.E., Jaeger U., Verhoef G., Schwenkglenks M., Bacon P. (2012). The need for improved neutropenia risk assessment in DLBCL patients receiving R-CHOP-21: Findings from clinical practice. Leuk. Res..

[42] Pettengell R., Bosly A., Szucs T.D., Jackisch C., Leonard R., Paridaens R., Constenla M., Schwenkglenks M., Impact of Neutropenia in Chemotherapy-European Study Group (INC-EU) (2009). Multivariate analysis of febrile neutropenia occurrence in patients with non-Hodgkin lymphoma: Data from the INC-EU Prospective Observational European neutropenia Study. Br. J. Haematol..

[43] Kuderer N.M., Dale D.C., Crawford J., Cosler L.E., Lyman G.H. (2006). Mortality, morbidity, and cost associated with febrile neutropenia in adult cancer patients. Cancer.

