# Supplementary material for: Predictive Model for Occurrence of Febrile Neutropenia after Chemotherapy in Patients with Diffuse Large B-Cell Lymphoma: A Multicenter, Retrospective, Observational Study
Source: Hematol Rep. 2024 Feb 7;16(1):76–88. doi: 10.3390/hematolrep16010008 (PMC10885064; doi:10.3390/hematolrep16010008)
Supplement: Supplementary file 1 [file hematolrep-16-00008-s001.zip › hematolrep-2710256-supplementary.pdf]

hematology reports

Article

Predictive model for occurrence of febrile neutropenia after chemotherapy in patients with diffuse large B-cell lymphoma: a multicenter, retrospective, observational study

Supplementary Material

Table S1. Univariate analysis of factors associated with febrile neutropenia incidence during any cycle for patients treated with the R-CHOP-like regimen.

	Developed FN during any cycle *n* = 64	Did not develop FN during any cycle *n* = 130	p-value
Age ≥ 65 years	47 (73%)	91 (70%)	0.62
Sex: male	38 (59%)	71 (55%)	0.53
Comorbidity			
Diabetes	10 (16%)	22 (17%)	0.82
Chronic kidney disease	5 (8%)	5 (4%)	0.24
Cardiac disease	12 (19%)	21 (16%)	0.65
Pulmonary disease	2 (3%)	2 (2%)	0.46
Malignancy	8 (12%)	16 (12%)	0.97
Baseline laboratory data			
WBC < 3.5 × 10^9 /L	7 (11%)	15 (12%)	0.9
Hemoglobin < 12.0 g/dL	33 (52%)	59 (45%)	0.42
Platelet < 100 × 10^9 /L	14 (22%)	10 (8%)	0.005
ANC < 1.5 × 10^9 /L	2 (3%)	6 (5%)	0.62
ALC < 0.7 × 10^9 /L	24 (38%)	18 (14%)	< 0.001
T-Bil > 1.0 g/dL	9 (14%)	18 (15%)	0.92
Albumin < 3.5 g/dL	36 (58%)	35 (27%)	< 0.001
LD > 222 IU/L	43 (68%)	66 (51%)	0.025
CRP > 10 mg/dL	5 (8%)	5 (4%)	0.29
eGFR < 60 mL/min/1.73 m^2	22 (34%)	30 (23%)	0.095
sIL2R > 2000 U/mL	37 (58%)	35 (27%)	< 0.001
Ann Arbor stage: Advanced (III–IV)	49 (78%)	59 (45%)	< 0.001
Extranodal involvement	41 (64%)	61 (47%)	0.025
Bone marrow infiltration	16 (25%)	14 (11%)	0.01
Viral hepatitis status			

Positive hepatitis panel	18 (28%)	22 (17%)	0.07
HCV	9 (14%)	4 (3%)	0.004
HBV	13 (20%)	20 (15%)	0.39
HBsAg +	1 (2%)	2 (2%)	0.99
anti-HBs +	7 (11%)	14 (11%)	0.97
anti-HBc +	13 (20%)	15 (12%)	0.1

Abbreviations:
ALC, absolute lymphocyte count; ANC, absolute neutrophil count; anti-HBc, hepatitis B core antibody; anti-HBs, hepatitis B surface antibody; CRP, C-reactive protein; eGFR, estimated glomerular filtration rate; FN, febrile neutropenia; HBsAg, hepatitis B surface antigen; HBV, hepatitis B virus; HCV, hepatitis C virus; LD, lactate dehydrogenase; R-CHOP, cyclophosphamide, doxorubicin, vincristine, and prednisone with added rituximab; sIL2R, soluble interleukin-2 receptor; T-Bil, total bilirubin; WBC, white blood cell.

Table S2. Univariate analysis of factors associated with febrile neutropenia incidence during the first cycle among patients in the salvage therapy group.

	Developed FN during the first therapy cycle $n = 9$	Did not develop FN during the first therapy cycle $n = 43$	p-value
Age ≥ 65 years	7 (78%)	29 (67%)	0.54
Sex: male	8 (89%)	29 (67%)	0.2
Comorbidity			
Diabetes	1 (11%)	3 (7%)	0.67
Chronic kidney disease	0 (0%)	4 (9%)	0.34
Cardiac disease	1 (11%)	4 (9%)	0.87
Pulmonary disease	0 (0%)	1 (2%)	0.64
Malignancy	2 (22%)	5 (12%)	0.4
Baseline laboratory data			
WBC < 3.5 × 10^9 /L	1 (11%)	6 (14%)	0.82
Hemoglobin < 12.0 g/dL	3 (33%)	19 (44%)	0.55
Platelet < 100 × 10^9 /L	2 (22%)	6 (14%)	0.53
ANC < 1.5 × 10^9 /L	1 (11%)	0 (0%)	0.027
ALC < 0.7 × 10^9 /L	3 (33%)	9 (21%)	0.42
T-Bil > 1.0 g/dL	0 (0%)	8 (20%)	0.17
Albumin < 3.5 g/dL	6 (67%)	10 (24%)	0.012
LD > 222 IU/L	7 (78%)	31 (72%)	0.73
CRP > 10 mg/dL	1 (12%)	2 (5%)	0.41
eGFR < 60 mL/min/1.73 m^2	2 (22%)	7 (16%)	0.67
sIL2R > 2000 U/mL	7 (78%)	18 (42%)	0.05
Ann Arbor stage: Advanced (III–IV)	9 (100%)	35 (81%)	0.16
Extranodal involvement	8 (89%)	30 (70%)	0.24

Bone marrow infiltration	2 (22%)	12 (28%)	0.73
Viral hepatitis status			
Positive hepatitis panel	4 (44%)	6 (14%)	0.035
HCV	4 (44%)	6 (14%)	0.035
HBV	1 (11%)	1 (2%)	0.21
HBsAg +	0 (0%)	2 (5%)	0.51
anti-HBs +	2 (22%)	4 (9%)	0.27
anti-HBc +	3 (33%)	3 (7%)	0.024

Abbreviations:
ALC, absolute lymphocyte count; ANC, absolute neutrophil count; anti-HBc, hepatitis B core antibody; anti-HBs, hepatitis B surface antibody; CRP, C-reactive protein; eGFR, estimated glomerular filtration rate; FN, febrile neutropenia; HBsAg, hepatitis B surface antigen; HBV, hepatitis B virus; HCV, hepatitis C virus; LD, lactate dehydrogenase; sIL2R, soluble interleukin-2 receptor; T-Bil, total bilirubin; WBC, white blood cell.

Table S3. Univariate analysis of factors associated with febrile neutropenia incidence during any cycle among patients in the salvage therapy group.

	Developed FN during any cycle<br>$n = 36$	Did not develop FN during any cycle<br>$n = 16$	p-value
Age ≥ 65 years	25 (69%)	11 (69%)	0.96
Sex: male	27 (75%)	10 (62%)	0.36
Comorbidity			
Diabetes	2 (6%)	2 (12%)	0.39
Chronic kidney disease	4 (11%)	0 (0%)	0.17
Cardiac disease	4 (11%)	1 (6%)	0.58
Pulmonary disease	0 (0%)	1 (6%)	0.13
Malignancy	5 (14%)	2 (12%)	0.89
Baseline laboratory data			
WBC < 3.5 × 10^9 /L	7 (19%)	0 (0%)	0.058
Hemoglobin < 12.0 g/dL	16 (44%)	6 (38%)	0.64
Platelet < 100 × 10^9 /L	7 (19%)	1 (6%)	0.22
ANC < 1.5 × 10^9 /L	1 (3%)	0 (0%)	0.5
ALC < 0.7 × 10^9 /L	11 (31%)	1 (6%)	0.055
T-Bil > 1.0 g/dL	6 (18%)	2 (12%)	0.61
Albumin < 3.5 g/dL	12 (34%)	4 (25%)	0.51
LD > 222 IU/L	27 (75%)	11 (69%)	0.64
CRP > 10 mg/dL	3 (9%)	0 (0%)	0.24
eGFR < 60 mL/min/1.73 m^2	7 (19%)	2 (12%)	0.54
sIL2R > 2000 IU/L	21 (58%)	4 (25%)	0.026
Ann Arbor stage: Advanced (III–IV)	30 (83%)	14 (88%)	0.7
Extranodal involvement	26 (72%)	12 (75%)	0.83
Bone marrow infiltration	11 (31%)	3 (19%)	0.38

Viral hepatitis status			
Positive hepatitis panel	7 (19%)	3 (19%)	0.95
HCV	7 (19%)	3 (19%)	0.95
HBV	2 (6%)	0 (0%)	0.34
HBsAg +	0 (0%)	2 (12%)	0.031
anti-HBs +	5 (14%)	1 (6%)	0.43
anti-HBc +	5 (14%)	1 (6%)	0.43

Abbreviations:
ALC, absolute lymphocyte count; ANC, absolute neutrophil count; anti-HBc, hepatitis B core antibody; anti-HBs, hepatitis B surface antibody; CRP, C-reactive protein; eGFR, estimated glomerular filtration rate; FN, febrile neutropenia; HBsAg, hepatitis B surface antigen; HBV, hepatitis B virus; HCV, hepatitis C virus; LD, lactate dehydrogenase; sIL2R, soluble interleukin-2 receptor; T-Bil, total bilirubin; WBC, white blood cell.

Table S4. Multivariate logistic regression analysis of risk factors associated with febrile neutropenia incidence during any cycle among patients treated with the R-CHOP-like regimen.

				$n = 191$
	Odds ratio [95% CI]	β coefficient [95% CI]	Score	p-value
ALC < 0.7 × 10^9 /L	2.61 [1.18–5.75]	0.96 [0.17–1.75]	1	0.018
Alb < 3.5 g/dL	2.33 [1.16–4.67]	0.85 [0.15–1.54]	1	0.017
Ann Arbor stage: Advanced (III–IV)	3.32 [1.60–6.89]	1.20 [0.47–1.93]	1	0.001

Abbreviation: Alb, albumin; ALC, absolute lymphocyte count; CI, confidence interval; R-CHOP, cyclophosphamide, doxorubicin, vincristine, and prednisone with added rituximab.

Table S5. Bootstrap validation of the predictive model for febrile neutropenia occurrence during any cycle among patients treated with the R-CHOP-like regimen and comparison with the original model.

			$n = 191$
	Logistic regression β coefficient [95% CI]	Bootstrapped β coefficient [95% CI]	Score
ALC < 0.7 × 10^9 /L	0.96 [0.17–1.75]	0.98 [0.12–1.79]	1
Alb < 3.5 g/dL	0.85 [0.15–1.54]	0.88 [0.15–1.54]	1
Ann Arbor stage: Advanced (III–IV)	1.20 [0.47–1.93]	1.21 [0.44–1.97]	1

Abbreviation: Alb, albumin; ALC, absolute lymphocyte count; CI, confidence interval; R-CHOP, cyclophosphamide, doxorubicin, vincristine, and prednisone with added rituximab.

Table S6. Multivariate logistic regression of risk factors associated with febrile neutropenia incidence during the first cycle of chemotherapy among patients treated with the R-CHOP-like regimen, with a more detailed evaluation of the viral hepatitis status.

			$n = 191$

	Odds ratio [95% CI]	β coefficient [95% CI]	p-value
ALC < 0.7 × 10^9 /L	6.04 [2.39–15.23]	1.80 [0.87–2.72]	<0.001
sIL2R > 2000 U/mL	3.03 [1.19–7.70]	1.11 [0.17–2.04]	0.020
Extranodal involvement	3.05 [1.08–8.57]	1.11 [0.08–2.15]	0.035
HCV	5.26 [1.28–21.69]	1.66 [0.24–3.08]	0.022
anti-HBc +	3.11 [1.00–9.68]	1.13 [-0.001–2.27]	0.050

Abbreviations: ALC, absolute lymphocyte count; anti-HBc, hepatitis B core antibody; HCV, hepatitis C virus; CI, confidence interval; R-CHOP, cyclophosphamide, doxorubicin, vincristine, and prednisone with added rituximab; sIL2R, soluble interleukin-2 receptor.

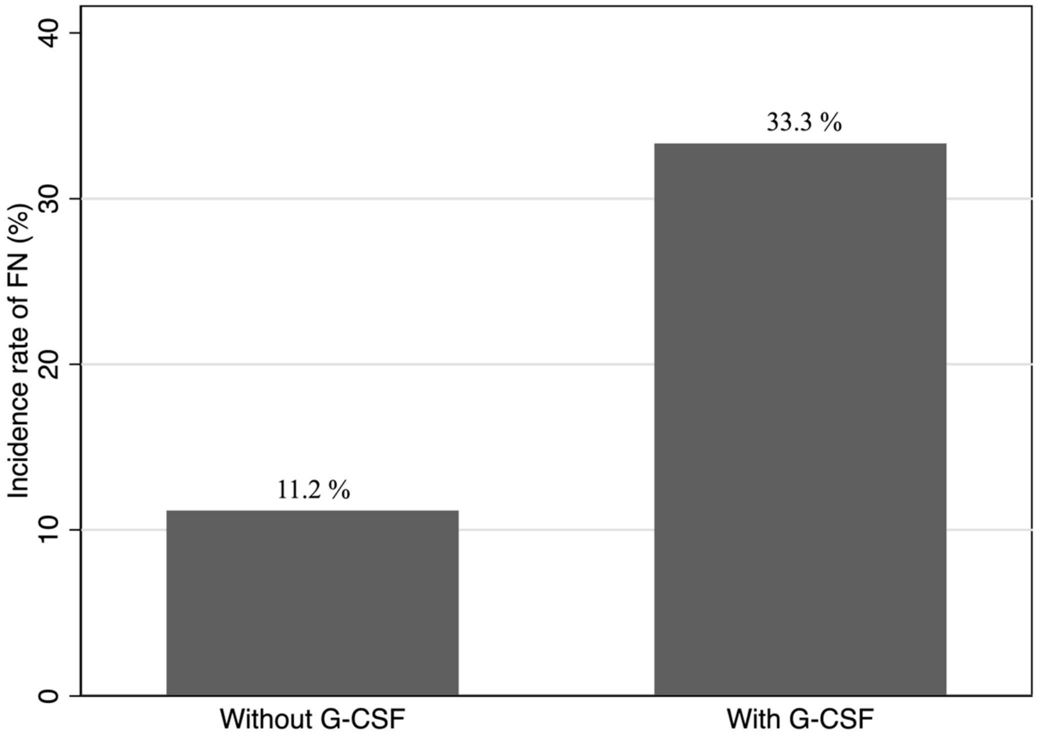

Figure S1. Differences in FN incidence between patients who received G-CSF during the first cycle of R-CHOP-like therapy and those who did not. Abbreviations: FN, febrile neutropenia; G-CSF, granulocyte colony-stimulating factor; R-CHOP, cyclophosphamide, doxorubicin, vincristine, and prednisone with added rituximab.

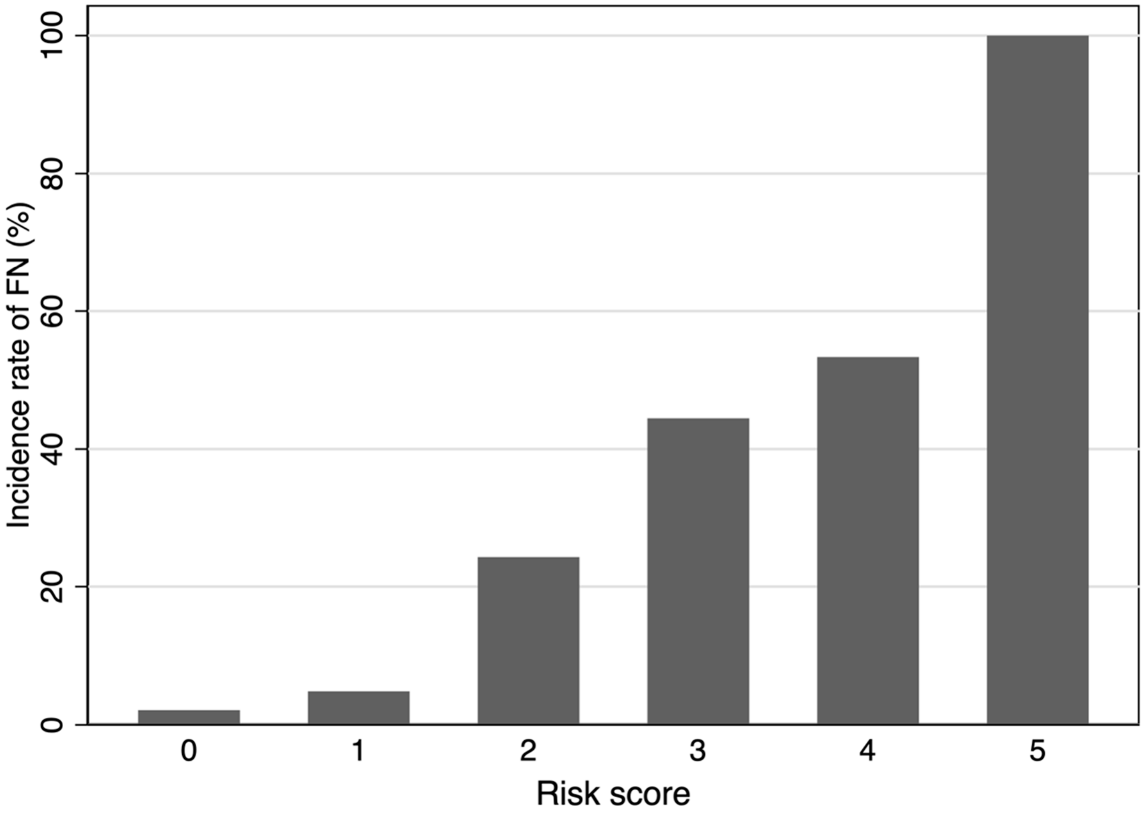

Figure S2. Incidence rate of FN during the first cycle of chemotherapy for patients treated with the R-CHOP-like regimen based on each prediction score. Abbreviations: FN, febrile neutropenia; R-CHOP, cyclophosphamide, doxorubicin, vincristine, and prednisone with added rituximab.
